# Exposure to volatile anaesthetics is not followed by a massive induction of single-strand DNA breaks in operation theatre personnel

**DOI:** 10.1007/s13353-015-0329-y

**Published:** 2015-12-21

**Authors:** Krzysztof Szyfter, Ireneusz Stachecki, Magdalena Kostrzewska-Poczekaj, Marcin Szaumkessel, Joanna Szyfter-Harris, Paweł Sobczyński

**Affiliations:** 1 Institute of Human Genetics, Polish Academy of Sciences, ul. Strzeszyńska 32, 60-479 Poznań, Poland; 2 Department of Anesthesiology and Intensive Therapy, University of Medical Sciences, Poznań, Poland; 3 Department of Dermatology, University of Medical Sciences, Poznań, Poland

**Keywords:** Volatile anaesthetics, Occupational exposure, DNA damage

## Abstract

Volatile anaesthetics such as halothane, isoflurane and others were expected to produce a health challenge for operation room personnel because of prolonged occupational exposure to anaesthetic gases. To estimate a molecular background of adverse health effects, a cohort of 100 exposed individuals was studied by the single-cell gene electrophoresis (comet assay) test. DNA lesions in lymphocytes of the exposed group did not differ significantly compared with non-exposed blood donors. Then, the exposed group was further divided according to job position. A highest level of DNA lesions was established in nurses but without significant difference compared with other groups. When a time period of exposure was taken into account, a tendency to cumulate DNA lesions was found only in the group of anaesthesiologists. A very weak genotoxic effect established in this study is discussed in relation to DNA repair, adaptative response and potential self-elimination of sensitive individuals.

## Introduction

Low-molecular-weight polyhalogenated (mostly fluorinated) hydrocarbons are volatile compounds used because of their properties as anaesthetic agents. Examples are halothane, isoflurane, sevoflurane, desflurane and other preferentially applicable as children hypnotics but also common in general anaesthesia (Ferstandig 1995; Reichle and Conzen 2003; Torri 2010). The activity of the volatile anaesthetics and the way of application results in a desired take-up by patients and non-intentional exposure of medical staff to list surgeons, anaesthesiologists, nurses and aids.

A proof for patients’ safety is being always required before the admission of novel drugs for use. However, in a given situation, patients are treated by drugs for hours/days, whereas medical staff could be exposed for tens of years. This is the case for operating theatre personnel inhaling volatile anaesthetics in their daily routine. Adverse health effects including reproduction malfunction (Allweiler and Kogan 2013; Boivin 1997; Lawson et al. 2012), hepatotoxicity (Stock and Strunin 1985), neurotoxicity and teratogenicity were reviewed by Byhahn et al. (2001). Malfunction of reproduction was also studied in respect to the generation of congenital anomalies. Estimation of a number of congenital anomalies in the children of over 15,000 females occupationally exposed to anaesthetic gases has revealed a potential exposure–response relationship (Teschke et al. 2011).

A discovery of health consequences of a prolonged exposure to anaesthetics was followed by numerous studies addressing the subcellular and molecular background of health damaging activity of inhaled anaesthetics. It was shown that halothane, sevoflurane, desflurane and isoflurane are capable of generating DNA damage, shown as single-strand DNA breaks, pointing at halothane as having the highest genotoxic potential (Brozovic et al. 2011; Jałoszyński et al. 1999; Karabiyik et al. 2001; Szyfter et al. 2004). In parallel studies, the clastogenic activity of halogenated anaesthetics was demonstrated as chromosome damage, increase of sister chromatid exchanges and micronuclei formation with cell death as an endpoint, although the results were not fully consistent (Musak et al. 2013; Pasquini et al. 2001; Wiesner et al. 2008; Yang et al. 2008). Discordance of the published results, to some extent, is connected with a variety of experimental protocols exploring human and animal models, exposure in vitro or in vivo, and various combinations of anaesthetic agents in a concentration from low to high. Hence, still there is a need to estimate a real risk to personnel exposed to halogenated anaesthetics in terms of molecular epidemiology.

Another argument to deal with the molecular effects of occupational exposure was a finding of the Austrian/Bayern group dated from the late 1990s describing the concentration of N_2_O and halogenated anaesthetics in Polish operation rooms as exceeding that in Western Europe by several times (Wiesner et al. 2000). Since that time, a situation has become adjusted to European standards because of the extensive modernisation of equipment in Poland, but some anxiety still remains among anaesthesiologists (Kucharska and Wesołowski 2014).

## Materials and methods

The study was approved by the institutional ethics committee. Informed consent was obtained from each participant. The study group comprised 100 persons exposed occupationally to anaesthetics by being employed as anaesthesiologists, surgeons, nurses and medical aids (Table 1) in operating theatres at university and local hospitals in the Poznań region (Central Poland). The control group consisted of volunteer blood donors adjusted by characteristics to the study group. The details of the sampling and determination of the concentration of waste anaesthetic gases (N_2_O, halothane, isoflurane and sevoflurane) by infrared spectrophotometer Miran® (OMC ENVAG, Warsaw, Poland) have already been described (Szulc et al. 2004). Sampling for anaesthetics concentration was done at each hospital twice: at the beginning and at the end of the working day. The ambient concentration of individual anaesthetics that surpassed the allowed level (2 ppm/m^3^) was found in 0.06, 1.45 and 3.46 % of operating theatres for halothane, isoflurane and sevoflurane, respectively (Szulc et al. 2004).

**Table 1:** Description of the study and control groups

	Study group	Controls
*N* (M/F)	100 (15/85)	100 (19/81)
Anaesthesiologist	26	–
Nurse	43	–
Scrub nurse	23	–
Medical aid	8	–
Duration of exposure	1–23 years	–
Smoking	24	n.d.
Drugs uptake:		
Cardiologic	4	3
Antihistamine	2	2
Endocrinologic	1	2
Analgesic	1	1
Neurologic	0	1
Antibiotics	1	0
Diabetes	0	1

Heparinised venous blood samples were collected from donors. Then, lymphocytes were separated with a standard method by centrifugation on Gradisol N. A suspension of lymphocytes derived from 100 μl in 1 ml of RPM 1640 medium was analysed for single-strand DNA breaks by an alkaline comet assay (single-cell gel electrophoresis), as previously described (Jałoszyński et al. 1999), except that visual scoring was replaced by an Axiophot fluorescent microscope (Zeiss, Germany) with an IMAC-CCD S30 camera and an ISI 3 v 2.00 image analysis system (MetaSystems Hard & Software, Altlusheim, Germany). Exposure-induced and spontaneous strand breaks were measured as the total comet length indicating DNA migration. All samples were processed in duplicates. Median values were calculated for 100 comets per slide.

The data were transformed to ranks to estimate the statistical significance by the Mann–Whitney *U*-test with GraphPad Prism software. The results were considered significant when *p* < 0.05.

## Results

DNA fragmentation in peripheral blood lymphocytes derived from subjects exposed to anaesthetic gases and the control group estimated by the comet assay (Table 2) did not reveal significant differences (*p* = 0.1793).

**Table 2:** DNA fragmentation shown by comet length (μm)

Study group	*n*	Mean ± SD	Median
Exposed group	100	43.21 ± 8.00	42.28
Controls	100	41.57 ± 9.02	40.22

**Fig. 1:**
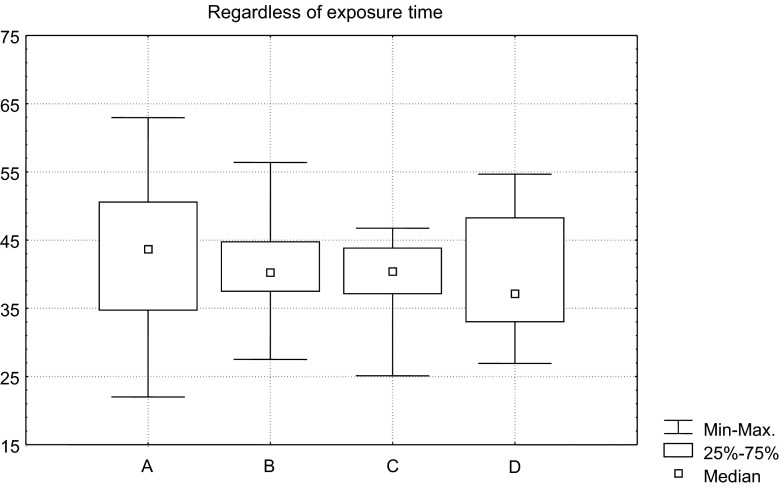
DNA fragmentation in the personnel of operation theatres according to job titles: **a** nurse; **b** anaesthesiologist and surgeon; **c** medical aid; **d** scrub nurse, regardless of exposure time

Then, a relationship between job position and genotoxicity of anaesthetic gases was estimated. Slight differences were found, pointing at nurses as the group with the highest detected genotoxic effect (Fig. 1). A large range of individual results within each group was established. The differences between the groups were almost ignorable.

**Fig. 2:**
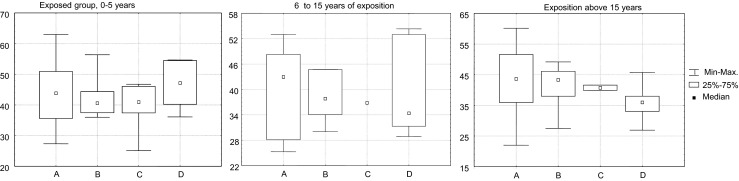
DNA fragmentation in relation to exposure time period in the exposed group subdivided according to job titles: **a** nurse; **b** anaesthesiologist and surgeon; **c** medical aid; **d** scrub nurse

Next, the exposure time was taken into account. We did not observe a considerable increase of genotoxicity towards exposure to volatile anaesthetics (Fig. 2). Noteworthy, a stable genotoxic effect in blood lymphocytes from nurses was different to a tendency to cumulate DNA lesions found in the group of anaesthesiologists (Table 3). The groups of anaesthesiologists and nurses outnumbered the other two groups, which makes these results more reliable.

**Table 3:** DNA fragmentation shown by comet length (μm)

Exposure (years)	Nurses	Anaesthesiologists
1–5	43.84 ± 9.88	40.60 ± 7.12
5–15	42.88 ± 10.47	37.82 ± 5.98
>15	43.66 ± 11.69	43.22 ± 7.26

## Discussion

A question of occupational safety in the operation theatre still remains open. A step forward was made when the admitted occupational doses were established by the National Institute for Occupational Safety and Health (USA) for N_2_O and halogenated anaesthetics at 25 ppm and 2 ppm, respectively. Other countries introduced their own regulations concerning concentration (Scapellato et al. 2008) and methodology recommended to estimate the risk factors (Kucharska and Wesołowski 2014). A list of threshold values introduced in the USA and some European countries can be found in a review paper by Byhahn et al. (2001).

A lack of genotoxic effect in the personnel of operating theatres was established in our study (general staff, anaesthesiologists) and a weak genotoxicity found in nurses is in opposition to the majority of findings reported by other researchers (Chandrasekhar et al. 2006; Musak et al. 2013; Rozgaj et al. 2009; Wiesner et al. 2008). On the other hand, there have been published reports on the similarities between anaesthetic exposed versus unexposed concerning the rate of sister chromatid exchanges (Krause et al. 2003; Lamberti et al. 1989; Pasquini et al. 2001) or the formation of micronuclei (Wiesner et al. 2008). Next, the review paper by Byhahn et al. (2001) provides further support for our findings, citing a large group of papers describing negative results concerning an effect of halogenated anaesthetics on DNA. To some extent, the differences in results could be explained by the variation of anaesthetic gases applied in hospitals, a large range and period of exposure, the size of study group and a variety of laboratory techniques applied. An impact of a dose of inhaled anaesthetics on molecular effects has already been pointed out (Wiesner et al. 2001).

In general, a measured genotoxicity results from a balance between the formation of DNA lesions and opposite processes, including lesions removal by DNA repair and elimination of damaged cells in the course of apoptosis. Concerning the genotoxic activity of halogenated anaesthetics, Jałoszyński et al. (1999) demonstrated DNA repair processing of lesions induced in vitro by halothane and isoflurane. The same study indicated cell necrosis replacing apoptosis when cells were treated with a high concentration of halothane. Another proof for a protective role of DNA repair and apoptosis was established by Alleva et al. (2003) in lymphocytes of orthopaedic patients under general anaesthesia.

The studied genotoxicity being poorly correlated with the time period of exposure to anaesthetic gases requires to consider at least a few confounders.

First, the studies on occupational health have shown that a part of employees’ response to work-related health risk is tending to move to another workplace. Early health effects (headache, nausea, fatigue) could be a part of the motivation to change an employment recognised as a safe one (Bonassi and Au 2002). Such self-elimination, known as healthy worker selection, results in an incomparability of ‘young’ and ‘old’ workers (Jones et al. 2013; Li and Sung 1999). In the given case, volatile anaesthetics could be tolerated better in the self-selected group working for a long time. A background of selectivity bias could be further extended on polymorphic gene variants responsible for xenobiotic processing in human cells. A relationship between the genotype distribution of genes coding detoxifying and DNA repair enzymes and DNA lesions was shown by Pavanello et al. (2005). An increased level of DNA adducts was found in coke oven workers exposed to polycyclic aromatic hydrocarbons being, at the same time, carriers of genotypes associated with low DNA repair capacity. Another example is the association of DNA damage with workers’ genetic constitution. The study concerns a risk of head and neck cancer in subjects exposed to polycyclic aromatic hydrocarbons. It has been shown that an exposure of over 10 years could be compensated by a protective variant of the DNA repair gene *XRCC1* (Khlifi et al. 2014). In line with the latter finding, there is a publication describing cytogenetic risk in coke oven workers exposed to polycyclic aromatic hydrocarbons. It was found that a high number of chromosome alterations and micronucleus formations could be reduced by polymorphisms of the DNA repair gene *XRCC1* (Sureshkumar et al. 2013). To the best of our knowledge, studies on the genetic modulation of anaesthetics tolerance are rare and concern only patients but not occupational exposure.

Further, a low genotoxic effect could be explained, to some extent, by adaptative response, which means cells better resist the damaging effects of toxic agents within a prolonged exposure (Fabiszewski and Skrzydlewska 1996). An adaptative response could be used to explain the results of the study on DNA damage in young medical residents of operating rooms exposed to isoflurane, desflurane and N_2_O. A growing increase of DNA damage shown by the comet assay was observed within 18 months, and then to drop at the 22 months control time (Costa Paes et al. 2014).

Lastly, it should be mentioned that, within the period of our studies, an intensive renovation of anaesthesiology equipment and scavenging systems took place in Poland that could be followed by exposure reduction (Kucharska and Wesołowski 2014; Kupczewska-Dobecka and Soćko 2006; Szulc et al. 2004).

To conclude, the current study demonstrates that an exposure to anaesthetic gases with a moderate genotoxic activity could be compensated in operating room personnel by such genetic factors as an efficient DNA repair, protective distribution of polymorphic gene variants, adaptative response and self-elimination of sensitive individuals.
